# Transforming tuberculosis (TB) service delivery model in China: issues and challenges for health workforce

**DOI:** 10.1186/s12960-019-0420-2

**Published:** 2019-11-12

**Authors:** Ziyue Wang, Weixi Jiang, Yuhong Liu, Lijie Zhang, Anna Zhu, Shenglan Tang, Xiaoyun Liu

**Affiliations:** 1 China Center for Health Development Studies, Peking University, No.38 Xueyuan Road, Haidian District, Beijing, 100191 China; 2 Department of Health Policy and Management, School of Public Health, Peking University Health Science Center, No.38 Xueyuan Road, Haidian District, Beijing, 100191 China; 3 Global Health Research Center, Duke Kunshan University, No. 8 Duke Avenue, Kunshan, 215316 Jiangsu Province China; 4 Beijing Chest Hospital, Capital Medical University, No. 97 Ma Chang, Tongzhou District, Beijing, 101149 China; 5 Clinical Centre on Tuberculosis, Chinese Center for Disease Control and Prevention, No. 97 Ma Chang, Tongzhou District, Beijing, 101149 China

**Keywords:** Tuberculosis, Health system, Service delivery model, Vertical approach, Integrated approach

## Abstract

**Background:**

China’s TB control system has been transforming its service delivery model from CDC (Centers for Disease Control and Prevention)-led model to the designated hospital-led model to combat the high disease burden of TB. The implications of the new service model on TB health workforce development remained unclear. This study aims to identify implications of the new service model on TB health workforce development and to analyze whether the new service model has been well equipped with appropriate health workforce.

**Methods:**

The study applied mixed methods in Zhejiang, Jilin, and Ningxia provinces of China. Institutional survey on designated hospitals and CDC was conducted to measure the number of TB health workers. Individual questionnaire survey was conducted to measure the composition, income, and knowledge of health workers. Key informant interviews and focus group discussions were organized to explore policies in terms of recruitment, training, and motivation.

**Results:**

Zhejiang, Jilin, and Ningxia provinces had 0.33, 0.95, and 0.47 TB health professionals per 10 000 population respectively. They met the national staffing standard at the provincial level but with great variety at the county level. County-designated hospitals recruited TB health professionals from other departments of the same hospital, existing TB health professionals who used to work in CDC, and from township health centers. County-designated hospitals recruited new TB health professionals from three different sources: other departments of the same hospital, CDC, and township health centers. Most newly recruited professionals had limited competence and put on fixed posts to only provide outpatient services. TB doctors got 67/100 scores from a TB knowledge test, while public health doctors got 77/100. TB professionals had an average monthly income of 4587 RMB (667 USD). Although the designated hospital had special financial incentives to support, they still had lower income than other health professionals due to their limited capacity to generate revenue through service provision.

**Conclusions:**

The financing mechanism in designated hospitals and the job design need to be improved to provide sufficient incentive to attract qualified health professionals and motivate them to provide high-quality TB services.

## Introduction

China has the world’s third largest TB epidemic. Eight hundred eighty-nine thousand new TB cases were detected in 2017, among which over 8% had multi-drug-resistant TB (MDR-TB), likely due to previously poor TB treatment in hospitals [1]. TB and MDR-TB cause great financial burden to patients and their family, which could in turn reduce patients’ adherence to treatment, especially for the poor [2, 3]. To tackle the high TB burden, China needs a strong TB control system of good quality and high efficiency.

China’s TB control system has been experiencing significant transition. China has developed a TB control system based on the Centers for Disease Control and Prevention (CDC) from early 1950s. In each county, a TB section within CDC or a separate TB dispensary was established to provide outpatient services and public health services. This CDC-led model was a unique design to combine both clinical and public health services within one institution [4]. While this model played a significant role in China’s TB control program, it has been facing great challenges. The shortage of qualified human resources for health (HRH) and limited clinical expertise and laboratory capacity in CDC cannot meet the increasing needs of TB care due to the high prevalence of MDR-TB and TB patients with co-morbidity [5]. In order to address these challenges, China started a new service delivery model in 2011, transferring the function of TB diagnosis and treatment from CDC to designated hospitals. CDC are responsible for TB control planning, supervision, and health education. Primary health care facilities are responsible for case management and patient referrals [6]. The new service delivery model was expected to improve the quality of TB health services. A vast majority of the Chinese provinces have now integrated the vertical TB control program into a general health service delivery system. However, a few provinces (like Jilin) have still maintained the old program.

Constraints on human resources for health (HRH), including deficits in number, unbalanced distribution, and limited capacity of health workers, have been reported as the main barriers to achieving global TB control target [7, 8]. Yet, data on HRH for TB control are rarely available and reliable in high-burden countries [9]. Whether the newly transformed TB service delivery model can achieve its objectives will largely depend on a TB health workforce with sufficient number, right knowledge and skills, and proper motivation. Zou and his colleagues revealed that the doctors from designated hospitals had better capacity to treat TB patients than CDC and TB dispensaries were the strongest rationale for integration [10]. Yet, the research questions of whether the new service delivery model can attract a sufficient number of TB health workers and whether the newly recruited staff at designated hospitals have proper knowledge and skill and are properly motivated to provide TB-related services remain unanswered. This study aims to identify implications of the new service model on TB health workforce development and to analyze whether the new service model has been well equipped with appropriate health workforce.

## Methods

### Study design and site selection

This study is part of a Gates Foundation-supported TB control program. Three provinces were selected for the study. Zhejiang represents the eastern developed area, Jilin is in less-developed central areas, and Ningxia is from the least-developed western area. For each province, a city with a higher GDP per capita and a city with a lower GDP per capita were selected. Then, we selected two counties in each city. For the county level, a county with a medium to high per capita GDP in each city and a county with a lower per capita GDP were selected. The total number of new and relapse TB patients registered should be more than 200 in 2014. If no county met this standard, the county with the highest number of registered TB patients was selected. The sample institutions included provincial-designated hospital/CDC, city-level-designated hospital/CDC, and county-level-designated hospital/CDC.

The study applied mixed methods to answer the research questions. Institutional survey on designated hospitals and CDC was conducted to measure the number of TB-related health workers. Individual questionnaire survey with TB health workers was conducted to measure the composition of health workers, their income, and knowledge. Key informant interviews with health managers and focus group discussions (FGDs) with TB health workers were organized to explore policies in terms of recruitment, training, and motivation [11]. In addition, relevant policy documents were collected to analyze TB staffing standards.

### Data collection

#### Questionnaire survey

An institutional survey questionnaire was sent to all CDC and TB-designated hospitals in the three provinces to investigate the HRH situation in these institutions. A second-round inquiry was carried out when the completed and returned questionnaire was found to have missing items or incorrect responses.

TB-related health workers who were on duty on the day of field work were invited to fill a survey questionnaire. Survey contents included their education background, workload, training, and income level. Questions in relation to TB diagnosis, treatment, and disease management were prepared for clinical doctors and public health doctors to answer, in order to measure their knowledge level. The knowledge questions were prepared by national TB experts. Research team members were present during the survey for quality control. In total, 281 TB-related health workers completed the questionnaire survey.

#### Key informant interviews and focus group discussions (FGDs)

Key informant interviews were conducted with health policy makers at the provincial, prefecture, and county levels and managers of CDC and designated hospitals. Interview topic guides included issues on staff recruitment, in-service training, and financial incentives. In each designated hospital, a FGD was convened with 6–8 health workers consisting of doctors, public health workers, nurses, and spear test technicians. Discussion topics included their workload, income, and training. In total, participants for key informant interviews and FGDs included 21 policy makers, 15 CDC managers, 21 managers of designated hospitals, and 21 groups of health professionals at designated hospitals.

A purposive sampling method was applied to cover different types of participants. The participants were recruited by coordinators from provincial- and county-level CDC. The interviews and FGDs were conducted in a quiet meeting room or office room. A senior researcher conducted the interviews and FGDs as interviewer or facilitator, with a junior researcher as observer and notetaker. There was no other person present during the interview/FGD. All interviews and FGDs were tape-recorded with informed consent.

### Data process and analysis

Questionnaire survey data were double entered using Epidata 3.1 and were analyzed using SPSS 20.0. Descriptive statistics was applied to analyze the HRH situation. *T* test and chi-square test were applied when it was necessary to compare between different groups. Ordinary least square (OLS) regression was conducted to identify influencing factors of staffing level (number of TB health workers per 10 000 population). Qualitative data were transcribed and analyzed using a thematic framework approach in MAXQDA 12. The analytical framework was developed based on the topic guides and emerging issues from the interviews and FGDs.

For both quantitative and qualitative data, analysis was conducted around three dimensions. First, staffing of TB health services included number and composition of TB health workers and sources of TB health worker recruitment. The second dimension was about the health workers’ TB knowledge. The third was about health workers’ income and financial incentives.

## Results

### TB service delivery models in the three provinces

**Fig. 1:**
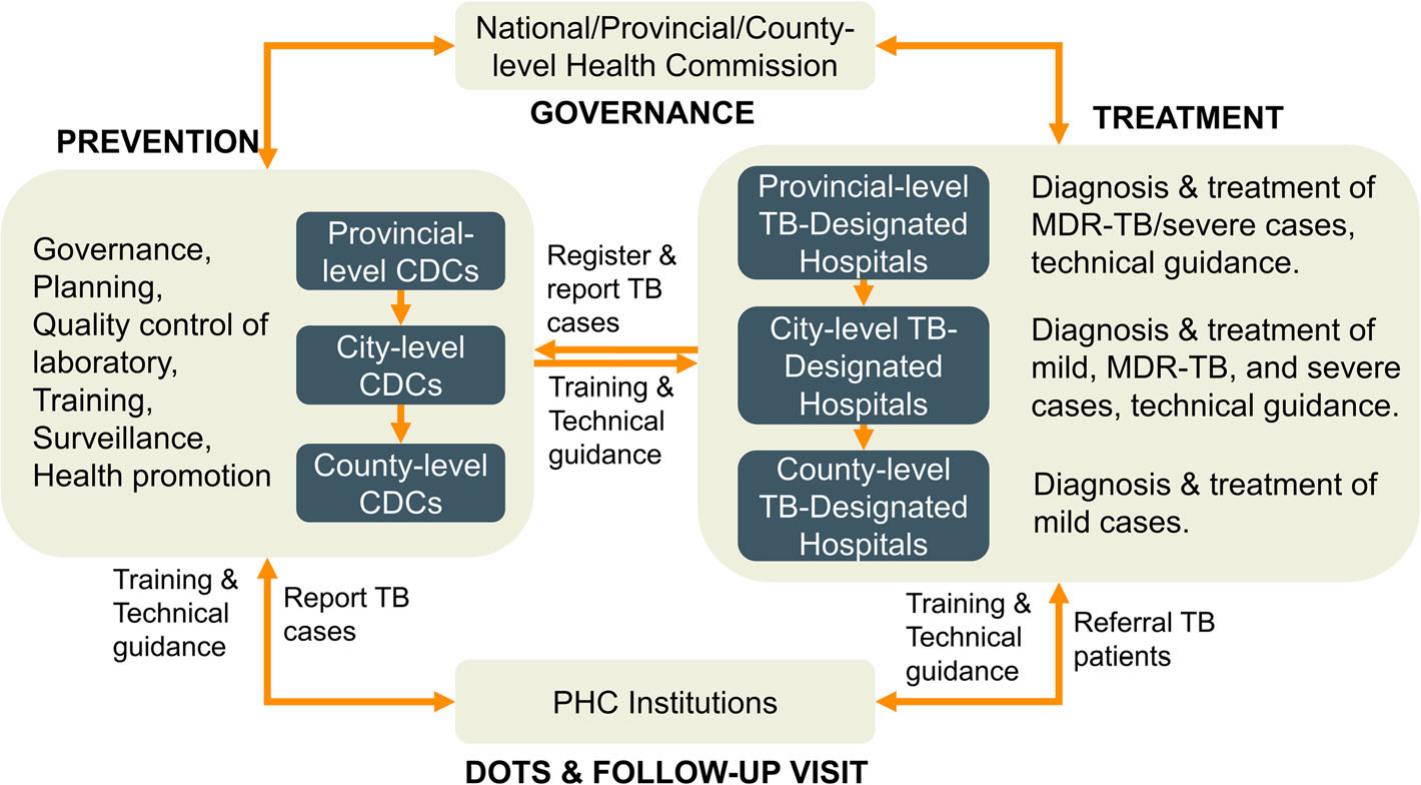
TB service delivery models in Zhejiang and Ningxia provinces. CDC, Center for Disease Control and Prevention; DOTS directly observed treatment, short course; MDR-TB, multi-drug-resistant tuberculosis; PHC primary healthcare

**Fig. 2:**
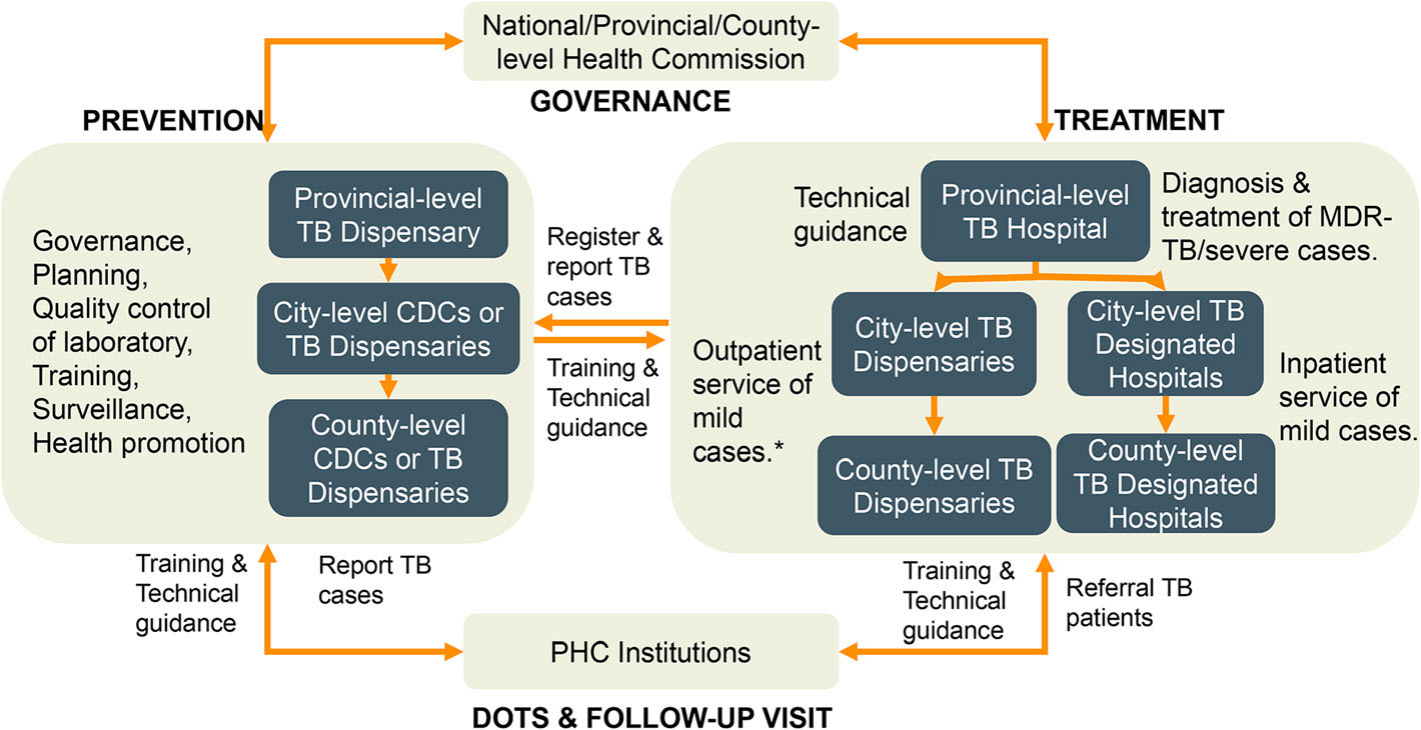
TB service delivery models in Jilin province

The three provinces were in different stages of service delivery model transformation. In Zhejiang, the service delivery model was completely transformed, i.e., TB service provision shifted from CDC to designated hospitals. County hospital as designated hospital provided outpatient and inpatient services for TB patients. County CDC was responsible for public health services for TB control. MDR-TB diagnosis and treatment services were provided at prefecture-level-designated hospital, a level higher than the county level but lower than the provincial level (Fig. 1). Jilin province still had the traditional CDC/TB dispensary model. Each county had a TB dispensary either within CDC or in separation. The TB dispensaries provided outpatient clinical services and public health services as well (Fig. 2). When a TB patient needs inpatient services, s/he could be referred to general hospitals. Ningxia applied a mixed model. At the county and prefecture levels, designated hospitals were developed to provide outpatient services for TB patients. But at the provincial level, there was a specialized TB hospital providing both clinical and public health services. Unlike in Zhejiang and Jilin, all MDR-TB patients in Ningxia received health services from provincial- rather than prefecture-level hospitals.

### Staffing of TB health services

The national TB control program guideline regulated in 2007 that each county should have at least eight TB health professionals including doctors, nurses, and public health workers. If the county has more than 400 000 people, one more health professional for every 50 000 people should be added. This standard was set before the transformation of the TB service delivery model. Table 1 shows that the three provinces had different levels of staffing. Jilin had 0.95 TB health professionals per 10 000 population, while Zhejiang only had 0.33. All three provinces met the national TB staffing norms. At the county level, 69.7% (62 out of 89) counties in Zhejiang, 98.3% (59 out of 60) in Jilin, and 70.0% (14 out of 20) in Ningxia can meet this staffing norm. The TB detection rates in Zhejiang, Jilin, and Ningxia in 2015 were 53.5, 47.1, and 39.2 per 100 000 population respectively, showing that the staffing level was not proportional to the TB prevalence in those regions.

**Table 1:** Number of TB health workers in three provinces in 2015

Province	TB detection rate (per 100 000 population)	Number of TB health workers per 10 000 population	Number of counties unmet the national TB health workforce standard (*N*, %)
Zhejiang	53.5	0.33	27/89 (30.34%)
Jilin	47.1	0.95	1/60 (1.67%)
Ningxia	39.2	0.47	6/20 (30.00%)

Data source: Institution survey. National TB health workforce standard is 0.2 TB health workforces per 10 000 population

Among the survey of 281 TB-related health workers, 61.3% were female, 72.7% aged between 30 and 50 years old, and 61.4% had a university degree. TB health workers in Jilin had a lower education level, with only 44.7% having a university degree, comparing to 79.2% in Zhejiang (Table 2). In the OLS regression holding the workload, provinces, population, and GDP per capita of the county fixed, the new service delivery model had 0.494 less TB health workers per 10 000 population than the old CDC-led model (Table 5 in Appendix).

**Table 2:** Composition of TB health workers (*N*, %)

	Zhejiang	Jilin	Ningxia	Total
Total	102	94	85	281
Level				
Provincial	20 (19.6)	10 (10.8)	16 (18.8)	46 (16.4)
Prefecture	33 (32.4)	31 (33.3)	29 (34.1)	93 (33.2)
County	49 (48.0)	52 (55.9)	40 (47.1)	141 (50.4)
Staff types				
Doctors	39 (38.2)	31 (33.0)	25 (29.4)	95 (33.8)
Nurses	21 (20.6)	19 (20.2)	16 (18.8)	56 (19.9)
Public health doctors	27 (26.5)	17 (18.1)	21 (24.7)	65 (23.1)
Technician	12 (11.8)	19 (20.2)	19 (22.4)	50 (17.8)
Others	3 (2.9)	8 (8.5)	4 (4.7)	15 (5.3)
Gender				
Male	45 (44.6)	34 (36.6)	29 (34.1)	108 (38.7)
Female	56 (55.4)	59 (63.4)	56 (65.9)	171 (61.3)
Age				
< 30	17 (17.0)	10 (10.8)	13 (15.3)	40 (14.4)
30–50	75 (75.0)	70 (75.3)	57 (67.1)	202 (72.7)
> 50	8 (8.0)	13 (14.0)	15 (17.6)	36 (12.9)
Education level				
Technical school and below	8 (7.9)	22 (23.4)	5 (5.9)	35 (12.5)
College (3 years)	13 (12.9)	30 (31.9)	30 (35.3)	73 (26.1)
University (≥ 5 years)	80 (79.2)	42 (44.7)	50 (58.8)	172 (61.4)
Professional title				
Primary	25 (24.8)	34 (36.2)	24 (28.2)	83 (29.6)
Middle	44 (43.6)	38 (40.4)	34 (40.0)	116 (41.4)
Senior	32 (31.7)	22 (23.4)	27 (31.8)	81 (28.9)

Data source: staff survey

Key informants from Zhejiang and Ningxia reported that there was usually only one health worker in county CDC in charge of TB control. County hospital as TB-designed hospital also only had one doctor in a TB clinic providing outpatient services for TB patients. In the wards, hospitals usually had more than one doctor in the department of infectious disease, providing inpatient services to patients diagnosed with TB and other infectious diseases. In Jilin where the traditional CDC model still dominated, TB dispensaries had more human resources. A FGD with TB health professionals revealed that one county TB dispensary in Jilin had 10 doctors, 12 nurses, 7 public health workers, and 11 other health professionals.

When the TB diagnosis and treatment service were transferred from CDC to designated hospitals, hospitals need to recruit TB doctors, nurses, and technicians, while CDC needed to re-deploy the TB health professionals who originally undertook these tasks. There were usually three sources for designated hospitals to recruit these cadres. The most common source of recruitment was from other departments of the same hospital. For example, in Zhongning County, Ningxia, a doctor was transferred from a pediatric department to become a TB doctor after some basic training.

During the transmission period, most of the TB health professionals who used to work in CDC were moved to other positions within CDC, but still some designated hospitals tried to recruit TB doctors from CDC to staff their newly established TB clinic. This became the second source of TB doctors for designated hospitals. Changshan County in Zhejiang and Zhongwei Prefecture in Ningxia applied this model.

If neither of the two sources could work, county hospitals could recruit health workers from township health centers to become TB-related health workers. In Tongxiang County, Zhejiang, the county hospitals recruited three doctors from township health centers. After several months’ in-service training, two became TB doctors and the other became a technician for smear test.

The old doctor we recruited from CDC had fixed post in the TB clinic. When he is retired, other doctors from the ward will have rotation to work between the TB clinic and the ward. Young doctors are reluctant to work in the TB clinic. (Manager of county designated hospital in Zhejiang)

### Health professionals’ knowledge of TB control

During the survey, TB clinical doctors and public health workers were asked to answer some pre-designed questions to measure their knowledge on TB control. Out of a total score of 100, clinical doctors got 67 in average, and public health workers got 77. Doctors in Jilin and Ningxia had significantly lower performance in the exam than those in Zhejiang (*p* < 0.05, Table 3).

**Table 3:** TB knowledge of health workers in three provinces

	Clinical doctors			Public health workers		
	*N*	Average score	*T* value	*N*	Average score	*T* value
Total	133	67	NA	81	77	NA
Zhejiang	50	84	Reference	29	83	Reference
Jilin	54	50	− 4.39*	29	71	− 2.54*
Ningxia	29	69	− 2.29*	23	78	− 1.59

*NA* not available

*p < 0.05

Key informants from designated hospitals revealed that they usually chose less competent doctors to staff the new TB clinic, with the justification that the TB clinic only provides basic free services which cannot raise revenues for the hospital. The new TB doctors in designated hospitals usually lack training with little clinical experiences, which consisted our findings in the data analysis.

### Income level and motivation policies

TB health workers were paid 4587 RMB Yuan (667 USD) per month for their work. Those in Ningxia and Jilin had much lower income than in Zhejiang. Health workers at the county level (4356 Yuan, 634 USD), female (4220 Yuan, 614 USD), younger than 30 years old (3227 Yuan, 470 USD), without a bachelor degree (4369 Yuan, 636 USD), and with primary professional title (3 678 Yuan, 535 USD) had lower monthly income than their counterparts (Table 4). Compared to 2014, the growth rate of average monthly wage for TB workers was 5.6% in 2015. 43.4% health workers reported their income were lower than the average level of their organization. The proportion was especially high among those < 30 years old (65.0%) and those with a primary professional title (61.4%).

**Table 4:** Monthly income of TB health workers

	*N*	Monthly income	*T* value	Annual growth rate	*T* value	Lower than average level (%)	Chi-square value
Total	278	4 536	NA	5.6	NA	43.4	NA
Province							
Zhejiang	100	5 815	Reference	6.5	Reference	57.0	Reference
Jilin	94	3 826	− 9.11*	4.5	− 1.13	35.1	10.85*
Ningxia	84	3 972	− 7.84*	6.4	− 0.04	39.8	8.10*
Level							
Provincial	46	5 108	Reference	1.5	Reference	28.3	Reference
Prefecture	92	4 720	− 1.30	6.1	1.58	47.3	5.12*
County	139	4 356	− 3.62*	7.0	1.91	47.5	5.70*
Staff types							
Doctors	94	4 993	Reference	5.1	Reference	46.8	Reference
Nurses	55	3 884	− 3.97*	6.7	1.76	58.2	0.69
Public health doctors	65	4 532	− 1.69	4.7	− 1.73	28.1	10.73*
Technician	49	4 480	− 1.72	5.0	− 0.04	42.9	0.95
Others	15	3 972	− 1.74	6.0	− 0.07	53.3	0.63
Gender							
Male	107	5 076	Reference	7.3	Reference	41.5	Reference
Female	170	4 220	− 4.02*	4.6	− 1.48	45.4	0.52
Age							
< 30	40	3 227	Reference	13.9	Reference	65.0	Reference
30–50	201	4 574	4.12*	4.6	− 3.65*	44.0	3.05*
> 50	35	5 361	5.23*	3.5	− 3.48*	25.7	9.03*
Education level							
Technical school and below	34	4 306	Reference	10.3	Reference	35.3	Reference
College (3 years)	73	4 398	0.67	5.2	− 1.68	46.6	0.79
University (≥ 5 years)	171	4 987	3.15*	4.7	− 2.14*	45.3	0.86
Professional title							
Primary	83	3 678	Reference	8.5	Reference	61.4	Reference
Middle	115	4 614	6.09*	4.6	− 1.68	43.0	6.29*
Senior	80	5 523	10.57*	4.6	− 1.40	28.8	18.80*

The average overall monthly physician salary in Zhejiang, Jilin, and Ningxia are 8 392 Yuan, 4 479 Yuan, and 5 006 Yuan, respectively (data source: Zhejiang, Jilin, and Ningxia Statistical Yearbook 2016)

*NA* not available

*p < 0.05

Key informant interviews showed that most designated hospitals had special financial incentive policies to motivate TB-related health workers. Designated hospitals set up higher proportion of revenue return to the TB department. In one hospital in Zhejiang, 19% of total service revenues were returned to the TB department as bonus incentive to motivate the TB health workers, while other clinical departments only got a 12% revenue return. Special allowances for TB health workers were another commonly used incentive. A daily rate of 9 RMB Yuan (1.3 USD) was provided as risk allowance to TB health workers at the infectious disease department.

Regardless of these incentive schemes towards TB health workers, they still reported lower income than their colleagues in other departments of the hospital.

We are a special department. Our department has higher consumption of supplies and therefore higher cost, but we have lower workload [to generate revenue]. Although our hospital had special financial policy to support our department, we still have the lowest income in the hospital. (TB health professional FGD in Zhejiang)

## Discussion

This study tried to analyze the implications of TB service delivery model on HRH development in three provinces of China. It found that Zhejiang province has 0.33 health workers per 10 000 population, less than Jilin (0.95) and Ningxia (0.47). TB clinical doctors showed limited TB knowledge compared to the public health workers. Although TB health professionals in the designated hospitals seemed to have higher income than their counterparts in CDC and TB dispensaries, they were still less motivated than their colleagues in other departments of the hospital.

China transformed its TB service delivery system from a vertical, disease-specific control system to a more integrated system. A TB control program integrated within wider health systems is believed to improve its performance [12, 13]. Previous studies confirmed that a more integrated system would benefit the service coverage and the treatment outcomes. Wei and his colleagues found that the new integrated TB service delivery model in China has shortened patient treatment pathways, improved quality, and reduced costs [14, 15].

This improvement of TB control outcomes in an integrated system largely depends on the availability of adequate workforce with appropriate competence and motivation. Adequate number of health professionals is a pre-requisite for effective TB control. The national TB control program has set a minimum staffing norm of 0.2 per 10 000 population. This was set 10 years ago, before the transformation of the TB service delivery model [16]. In the new model, staffing norms need to be updated accordingly [8]. This study found that although the total number of TB health professionals at the provincial level met the national TB health workforce standard, they are not evenly distributed. About 30–50% counties with the new service delivery model cannot meet the staffing norm, implying that they still need to increase the staff supply for TB control.

Comparison of TB health workforce between the three provinces shows that the traditional CDC-led model has more health workers than the new model. This is mainly because most of the existing TB health workers in CDC cannot move to the designated hospital, and they have to move to other disease control programs within CDC. Designated hospitals need to recruit and train new health workers to provide TB health services. To some extent, there is a waste of existing human resources in the transition of the TB service delivery model. Some studies also reported challenges of retaining and attracting skilled workforces during the TB service model transitions [10].

The quality of TB health professionals at designated hospitals is a key concern. Designated hospitals recruited TB doctors and other professionals from various sources (including other departments of the same designated hospital, CDC, or township health centers). Generally, the newly recruited health professionals are not highly qualified for TB diagnosis and treatment. Hospitals usually chose less-competent doctors from other departments to staff the TB department. Health professionals recruited from CDC and township health centers also had limited knowledge and skills in TB health services. This low qualification is reflected by their suboptimal performance in the knowledge test in the study. Because of the limited clinical qualification, designated hospitals usually put the newly recruited TB health professionals on fixed posts in the TB clinic. Most of them only provide TB outpatient services and do not have opportunity to practice in the inpatient wards and to treat patients with other diseases. This further limited their opportunity to improve their knowledge and skills. According to human resource management theory, exposure to inter-related multiple job tasks can enrich job experiences and help an employee learn new skills and reduce boredom [17, 18]. The job design of TB health professionals at designated hospitals needs improvement to maximize their job performance.

The financial management system in designated hospitals has important implications on TB health workforce development. Public hospitals in China largely depend on service revenues to cover personnel cost and operational cost [19]. Since most of TB diagnosis and treatment services are free services according to the national TB control program, the newly established TB department has limited capacity to generate revenues through service provision. When hospitals mobilize doctors from other departments to staff the TB department, usually less-qualified doctors were selected to fill the positions. Although designated hospitals introduce special financial incentives to attract and motivate TB health workers, they still only have a modest income and have low motivation and high turnover rate. In recent years, China is scaling up public hospital reform, with zero markup of medicines as a key policy lever [20]. The profit-driven service provision behavior may have considerable changes. The rapidly changing context of public hospital reform will have considerable implications on TB HRH development and service provision, which needs further observation and analysis.

The study has several limitations. First, the study depends on self-reported data to measure the HRH issues in TB control. Some of the information, especially regarding the self-reported monthly income, may be underreported. Second, without pre-transformation data on health workforce, the cross-sectional survey design may not imply a direct causal relationship between model transformation and HRH change. The differences between the three provinces may reflect a combination of various factors including social economic development status and service delivery model. This study tries to analyze whether the staffing level under the new service delivery model is appropriate to the TB prevalence and can meet the national TB staffing norms. In addition, the qualitative study also helps explain the challenges in health worker attraction and retention under the new model.

In conclusion, the reform of the TB service delivery system in China is in alignment with the global trend of integrating TB control into the overall health system to improve service quality and health outcomes. However, lack of qualified and properly motivated HRH could make the reform unsustainable. Local government and designated hospitals should develop special incentive programs to attract and retain TB health professionals. Job design of TB health professionals should be improved to allow them to have opportunities for better career development.

## Data Availability

The datasets used and analyzed during the current study are available from the corresponding author on reasonable request.

## Appendix

**Table 5:** Influencing factors of TB health workers per 10 000 population—OLS model

Variables	Dependent variable: TB health workers per 10 000 population	
Old system	0.494*** (0.141)	0.432*** (0.114)
Rural	− 0.115 (0.149)	0.180 (0.125)
Missing TB health workers	0.177 (0.222)	− 0.113 (0.151)
Log (inpatient visit)	− 0.133 (0.105)	0.041 5 (0.041 9)
Log (outpatient visit)	0.246** (0.093 6)	
Log (square)	− 0.013 2 (0.091 6)	− 0.006 04 (0.074 1)
Log (population)	− 0.463*** (0.132)	− 0.239*** (0.082 8)
Log (GDP per capita in 2015)	0.055 8 (0.121)	− 0.013 5 (0.099 3)
Constant	5.417*** (1.885)	3.490** (1.364)
Observations	101	138
*R*-squared	0.299	0.202

Old system: = 1 if the county still had the traditional CDC/TB dispensary model and = 0 if the service delivery model in the county was transformed

Rural: = 1 if the county is a rural area and = 0 if the county is an urban area

Missing TB health workers: = 1 if more than one hospital in the county did not report the number of their TB health workers

Log (outpatient visit)/log (inpatient visit)/log (square)/log (population)/log (GDP per capita in 2015): natural logarithm of the total number of the outpatient visit/inpatient visit/total square land area/permanent population/GDP per capita in 2015 within the county

Standard errors in parentheses. There are 138 counties in three provinces. However, the data of hospitalization rate of TB patients is available only in 101 counties

****p* < 0.01, ***p* < 0.05

## Abbreviations

CDC: Centers for Disease Control and Prevention
FGDs: Focus group discussions
HRH: Human resources for health
MDR-TB: Multi-drug-resistant TB
RMB: Renminbi
TB: Tuberculosis
USD: United States dollar
